# Correction to “AaSIZ1‐Mediated SUMOylation of AaMYB31 Positively Regulates Freezing Tolerance in *Actinidia arguta*”

**DOI:** 10.1111/pbi.70707

**Published:** 2026-06-25

Liu, L. Y. , L. Cao , N. Tang , W. M. Ren , Z. L. Zhu , R. Wang , M. H. Xiang , J. L. Sun , X. Zhang , F. Zhang , Y. Z. Lin , Y. T. Kong , Z. H. Jiang , L. H. Wang , Y. S. Liu , C. Zhang and P. P. Zheng 2026. “AaSIZ1‐Mediated SUMOylation of AaMYB31 Positively Regulates Freezing Tolerance in *Actinidia arguta*
.” Plant Biotechnology Journal 24: 3897–3921.41741012 10.1111/pbi.70620PMC13205662

In the above article, the authors would like to correct Figure 5D. In Figure 5D, the position between WT and *AaMYB31.2‐OE6* before freezing treatment was incorrect, and the correct Figure 5D is shown below.

**FIGURE 5D:**
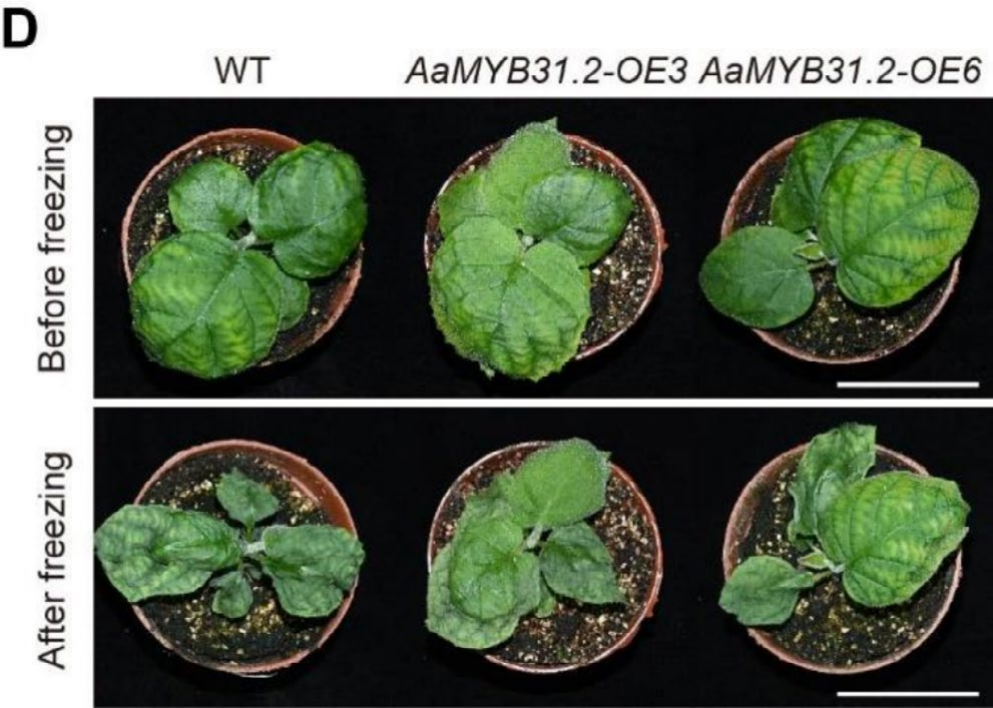
The freezing phenotype of *AaMYB31.2‐OE* transgenic *Actinidia chinensis* ‘Hongyang’ plants. Scale bars = 5 cm.

We apologize for this error.

